# Serum and salivary macrophage migration inhibitory factor in patients with oral squamous cell carcinoma

**DOI:** 10.3892/ol.2014.2513

**Published:** 2014-09-09

**Authors:** MARIANA BARBOSA DE SOUZA, OTÁVIO ALBERTO CURIONI, JOSSI LEDO KANDA, MARCOS BRASILINO DE CARVALHO

**Affiliations:** 1 Department of Radiology and Oncology, Medical School, University of São Paulo, São Paulo 01246903, Brazil; 2 Laboratory of Molecular Biology, Heliópolis Hospital, São Paulo 04231030, Brazil; 3 Department of Head and Neck Surgery and Otorhinolaryngology, Heliópolis Hospital, São Paulo 04231030, Brazil; 4 Department of Head and Neck Surgery, Padre Anchieta Teaching Hospital, ABC Medical School, São Bernardo do Campo 09715090, Brazil

**Keywords:** oral cancer, inflammation, macrophage migration inhibitory factor, serum biomarker, salivary biomarker

## Abstract

The overexpression of macrophage migration inhibitory factor (MIF) has been identified in a variety of tumors and the investigation of its molecular mechanisms in tumor progression is a key topic of research. The present study aimed to investigate MIF as a potential marker for disease control or recurrence, and to assess the association between serum and salivary MIF and the clinicopathological characteristics of patients with oral squamous cell carcinoma (OSCC). Serum and salivary samples were collected prior to and following the surgical treatment of 50 patients with OSCC. MIF concentrations were assessed by enzyme-linked immunosorbent assay and the adopted level of statistical significance was P<0.05. The results revealed that serum MIF concentrations were significantly reduced following tumor resection in OSCC patients. Furthermore, higher preoperative salivary MIF concentrations were observed in patients with larger tumors and in those who succumbed to the disease. In conclusion, high salivary and serological MIF concentrations were identified in patients with OSCC. Nevertheless, only serological MIF concentrations may be considered as a potential marker for the early detection of OSCC recurrence once the salivary levels, prior and following treatment, do not show any significant differences.

## Introduction

Oral squamous cell carcinoma (OSCC) is the most common type of cancer among head and neck neoplasms, and affects 275,000 individuals annually worldwide. In Brazil, the South American nation with the highest OSCC incidence, OSCC is the seventh most common type of cancer in the general population (1–3). Approximately 80% of OSCC cases are associated with tobacco and alcohol consumption; however, several other factors may also favor its development, including human papilloma virus infection and poor oral hygiene (1,4,5).

Although the oral cavity may be easily examined, OSCC is often diagnosed late, which contributes to poor overall survival (1,2). However, the identification of molecular biomarkers may improve existing clinical parameters for the development of novel diagnostic tools and treatment protocols, as well as aid in assessing prognosis (6). The identification of these biomarkers in the serum and saliva of OSCC patients is a promising and less invasive approach for the diagnosis, prognosis and assessment of disease status following therapy (7,8).

It is known that cancer can have a significant inflammatory component and, in certain cases, inflammation itself may trigger a malignant transformation. In other cases, such as in OSCC, inflammation is caused and modulated by genetic and epigenetic alterations induced by carcinogens, including tobacco and alcohol. The modulation of the inflammatory response contributes to tumor progression by increasing malignant cell proliferation and survival, stimulating neoangiogenesis and reducing antitumor immunity and tumor responses to therapies (9–12).

Cytokines are important components of the inflammatory cancer-associated process. Additionally, tumor cells often overexpress cytokines in order to modulate their microenvironment, which therefore indicates a potential role for cytokines as biomarkers and therapeutic targets (12). Macrophage migration inhibitory factor (MIF) is a pro-inflammatory cytokine that regulates the innate immune response and has been shown to be important in various autoimmune diseases, in addition to being involved in cell proliferation, cell survival, migration and metastasis in cancer (13–19). In addition, previous studies have revealed that high serum MIF concentrations are observed in patients with colorectal, prostate, colon and gastric cancers, when compared with healthy subjects (20–23). High serum MIF concentrations in patients with prostate, gastric and hepatocellular cancer were found to be associated with a poor prognosis (24–26).

In oral cancer, Kindt *et al* (27) demonstrated that, compared with normal tissues, MIF is overexpressed in tumors, indicating that this cytokine may contribute to tumor progression and the emergence of second primary tumors (27).

The aim of the present study was to assess the serum and saliva MIF concentrations in OSCC patients, prior to and following surgical treatment, and their correlation with clinicopathological characteristics. Serum and saliva MIF concentrations were also investigated as potential markers for disease control and recurrence in these patients.

## Materials and methods

### Study population

The study included 50 prospectively enrolled patients with primary OSCC who were treated at Heliópolis Hospital (Sao Paulo, Brazil) or the Padre Anchieta Teaching Hospital (Sao Paulo, Brazil) between 2011 and 2013. This study was approved by the ethics committees of Heliópolis Hospital, ABC Medical School (São Bernardo do Campo, Brazil) and the Medical School of the University of São Paulo (São Paulo, Brazil) and included only male patients with no history of autoimmune disease or prior cancer affecting any other anatomic areas, and to whom surgical treatment with or without postoperative radiotherapy and/or chemotherapy had been proposed. All patients provided written informed consent to participate. The clinicopathological characteristics of the patients are shown in Table I.

### Patient samples

Prior to (0–30 days) and following (20 days to 3 months) surgery, 8-ml whole blood samples and 5 ml of total, non-stimulated saliva were collected from each patient. The collections were performed between 9 and 10am. The patients were advised not to eat, drink, smoke or use oral hygiene products for at least 2 h prior to collection. Blood samples and saliva were cooled during transportation and were immediately centrifuged for 15 and 20 min, respectively, at 5031 × g and 4°C. Next, 0.8 μl of protease inhibitor cocktail (cat. no. P8340; Sigma-Aldrich, St. Louis, MO, USA) was added to each 400-μl aliquot of serum or saliva. The samples were then stored at −80°C until enzyme-linked immunosorbent assay (ELISA) analysis was performed. When required for the study, the samples were thawed at room temperature (18–25°C) and used immediately.

### ELISA

The MIF concentrations in the serum and saliva samples were assessed using an ELISA kit (Quantikine, cat. no. DMF00B; R&D Systems, Minneapolis, MN, USA) according to the manufacturer’s instructions.

The optical densities were determined using a microplate reader (ELX800; BioTek Instruments, Inc., Winooski, VT, USA) at a wavelength of 450 nm. To determine the standard curve, the original concentrations of various dilutions of recombinant human MIF were correlated with the corresponding optical densities.

The dilutions used for serum and saliva samples were 1:10 and 1:100, respectively. The samples exhibiting absorbances that were not included in the standard curve were diluted as required. The MIF concentrations in the samples were calculated according to the standard curve and presented as the mean of triplicates (ng/ml).

### Statistical analysis

The Shapiro-Wilk test was used to assess the normally distributed MIF concentrations in the serum and saliva samples. MIF concentrations in the samples prior to and following surgery were compared using the Wilcoxon matched-pairs signed-ranks test. The nonparametric Mann-Whitney U test was used to assess the associations between variables with two categories relative to the MIF concentrations in the serum or saliva. The nonparametric Kruskal-Wallis test was used for variables with three categories. P<0.05 was considered to indicate a statistically significant difference. STATA software, version 7.0 (StataCorp., College Station, TX, USA) was used to perform all statistical tests.

Novel indices were proposed for correlations with the quantity of tumor tissue. The lymph node index (LNI) was calculated as the sum of the number of metastatic lymph nodes and diameter (cm) of the largest positive lymph node. The general index (GNI) was defined as the sum of the LNI and the largest diameter (cm) of the primary tumor.

## Results

### MIF concentrations in serum and saliva samples prior to and following surgery

**Figure 1:**
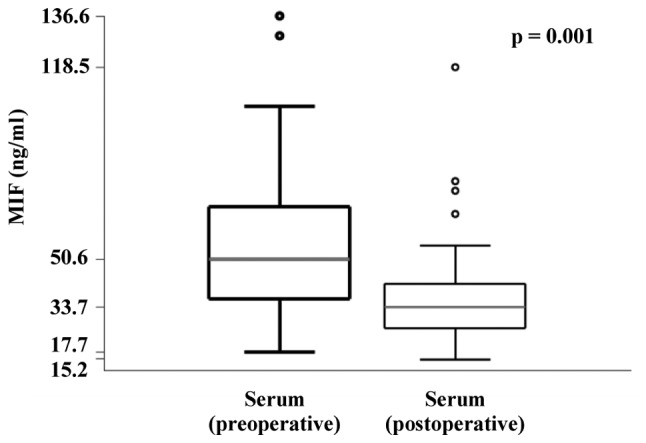
MIF concentrations in pre and post-operative serum samples of patients with oral squamous cell carcinoma. MIF, macrophage migration inhibitory factor.

The results revealed that the MIF concentration was significantly decreased in the postoperative serum samples (Fig. 1). Confirming this result, a statistically significant decrease in serum MIF concentrations was identified following surgery when the data were stratified by disease status following treatment (Table II). However, no significant differences in MIF concentration were identified between the saliva samples collected prior to and following surgery (data not shown).

### Associations between serum and saliva MIF concentrations and clinicopathological data

Correlations between the concentrations of MIF in serum and saliva, collected prior to and following surgical treatment, and clinicopathological data are shown in Table III.

The MIF concentrations in the preoperative saliva of patients was associated with tumor size; saliva concentrations were higher in patients with pT3 and pT4 stage tumors (P=0.001; Table III) and in patients with tumors >2.5 cm (P=0.020; Table IV). Consequently, more advanced disease stage, stages III and IV (P=0.032; Table III), and a high GNI (0.025; Table IV) were associated with increased MIF concentrations in the saliva samples collected prior to tumor resection. Salivary MIF concentrations prior to surgery varied according to surgical margin involvement (P=0.045; Table III).

In preoperative serum samples, the MIF concentrations were found to be significantly lower in patients with lymph node involvement (P=0.018; Table III). However, when comparing the LNI, patients with an LNI >3.5 exhibited higher MIF concentrations in preoperative serum samples (P=0.025; Table IV).

Table II shows that MIF concentrations in the preoperative saliva samples were higher in patients who succumbed to the disease than in surviving patients (P=0.023).

No significant associations were identified between the MIF concentrations in serum and saliva collected prior to surgery and perineural invasion, or tumoral inflammatory cell infiltration (Table III).

## Discussion

MIF has been considered to present an important link between inflammation and cancer due to its pro-inflammatory role, overexpression in various tumor tissues and interactions with pathways that aid tumor progression. Its molecular mechanisms involve, among others, the inhibition of p53, extracellular-signal-regulated kinase/mitogen-activated protein kinase and AKT/protein kinase B activation, and sustained hypoxia-inducible factor 1-α activation, all of which promote tumor cell proliferation, cell survival and tumor-associated neoangiogenesis (16,28–32). Under normal conditions, a variety of immune cells, as well as the pituitary gland and endothelial and epithelial cells of different organs, express MIF (15,33). Several studies have identified MIF overexpression in tumors when compared with healthy tissues, and high MIF concentrations were detected in the serum of cancer patients when compared with healthy controls. Therefore, these data indicate a potential function for this protein as a biomarker of neoplastic diseases (20–22,34–40).

In the present study, in order to evaluate MIF as a serological and salivary biomarker of OSCC, MIF concentration in pre- and postoperative serum and saliva samples of patients with OSCC was investigated. To avoid interference of uncontrolled variables when comparing the MIF concentrations in different individuals, we used samples collected from the same patient following tumor resection as controls. Since MIF overexpression is also associated with autoimmune diseases (41–43), as well as with previously mentioned polymorphisms in the gene promoter of *mif* (44,45), a comparison of samples of the same individual prior to and following tumor resection allowed control of these variables.

In this study, the serological MIF concentrations following tumor resection were significantly lower than those prior to tumor resection. This is consistent with the hypothesis that, since MIF is often overexpressed in tumors, it may also be detected at high levels in the serum of patients with OSCC. Considering the non-significant differences in MIF serological concentrations according to tumor size, this result indicates a possible function for MIF as a serological marker for OSCC detection, regardless of tumor extension. The serum MIF concentration was found to inversely correlate with lymph node involvement, in contrast to previous studies, which have reported that MIF induces the migration and invasion of tumor cells (38,46–49). However, with regard to LNI, which may present the total metastatic lymph node mass, the serological MIF concentration was higher in individuals with a higher LNI. The LNI was calculated to compare the MIF concentrations in serum and saliva with more representative data regarding the total quantity of regional tumor present in the patient, since the number of metastatic lymph nodes or the diameter of the largest metastatic lymph node alone was not sufficient.

The dual and complex role of MIF has been discussed in detail. A study of patients with head and neck squamous cell carcinoma revealed correlations between low and high tumoral immunohistochemical expression of MIF and poor survival, and between moderate expression and improved survival (50). In addition, in the same study, high MIF expression in the tumor was positively associated with lymph node involvement, whereas low and moderate expression was found to correlate with no regional metastasis. Verjans *et al* (51) revealed that cytoplasmic MIF expression in tumor tissues was associated with improved survival in breast cancer patients, indicating that intracellular MIF may inhibit cell proliferation and indicate a favorable prognosis, whereas extracellular tumor tissue-derived MIF may be pro-inflammatory and may be associated with an unfavorable prognosis (51). These results demonstrate that the function of MIF in the progression and prognosis of several malignancies remains controversial, and further studies are required to investigate its different mechanisms of action, particularly with regard to its origin (from healthy tissues or tumors) and location within the cell. Accordingly, the current study group is also investigating MIF expression in tumor tissue and surgical margins.

To the best of our knowledge, the present study is the first to investigate MIF levels in saliva samples. It was observed that high levels of this protein are present in pre- and postoperative saliva of OSCC patients. Considering that OSCC cells secrete proteins that are eluted into the saliva, possibly via direct contact, it was hypothesized that salivary MIF concentration would decrease following tumor resection. However, this was not observed. We hypothesized that this result may be due to the constitutive expression of MIF by endocrine, immune, and particularly epithelial cells that are in direct contact with the external environment and regulate host responses to infections and stress. Pathogen-associated molecular patterns and inflammatory cytokines, including tumor necrosis factor-α and interferon-γ, are potential inducers of MIF secretion by macrophages, and the vast microbiota present in the oral cavity may facilitate this process, thus maintaining a high MIF concentration in the saliva (15). In addition, the inflammation triggered by the operative wound healing process may have increased the MIF concentrations in the saliva samples. However, an interval of 20–30 days following surgery was selected for sample collection, and longer periods were not considered as following this period the referred patients began radiotherapy and chemotherapy treatment, which may have interfered with the analysis. The salivary MIF concentrations were significantly higher in patients with larger tumors and those at more advanced pathological stages, than in patients with smaller tumors and those at initial pathological stages. On the basis of these results, we hypothesize that salivary MIF may not originate exclusively from OSCC cancer cells and, therefore, may not present a reliable marker for tumor diagnosis. MIF may also originate from endocrine, immune, and epithelial cells, and this may contribute to tumor progression via the aforementioned mechanisms. Regarding disease control status and prognosis evaluation, higher salivary MIF concentrations were identified in patients who succumbed to the disease than in those who survived. However, the limited follow-up period of this study was not long enough to comprehensively evaluate survival. This result indicates a possible role for MIF in the prognosis of patients with OSCC; however, further studies are required to confirm this correlation.

As previously reported, MIF expression may be induced by epidermal growth factor (EGF) in breast cancer cells and also appears to be involved in the proliferative pathway activated by EGF (52). To date, the EGF receptor (EGFR) pathway is the most important pathway associated with OSCC development, and the investigation of the association between MIF and EGFR in OSCC may be extremely noteworthy. In addition, OSCC is an extremely heterogeneous disease; using MIF as a biomarker may be more useful when associated with other markers with known importance in OSCC development and prognosis, including other cytokines and proteins of the EGFR pathway.

In conclusion, the increased serological MIF concentrations in these patients prior to treatment observed in this study indicate a potential role for MIF as a biomarker for the early detection of OSCC recurrence. However, a long-term validation study is required, with a greater number of patients to evaluate serological MIF concentration in different disease statuses and during follow up or including MIF in a panel of markers.

## Figures and Tables

**Table I:** Clinicopathological characteristics of patients with OSCC (n=50).

Clinicopathological characteristics	OSCC frequency (%)
Age (years)	
Range	40–88
Median	56.5
Mean (standard deviation)	56.2 (8.3)
Ethnicity	
Caucasian	31 (62.0)
Non-caucasian	16 (32.0)
Not available	3 (6.0)
Smoking status	
Current smoker	42 (84.0)
Ex-smoker	8 (16.0)
Alcohol consumption	
Current drinker	29 (58.0)
Ex-drinker	20 (40.0)
Never	1 (2.0)
Postoperative RT	
Yes	22 (44.0)
No	28 (56.0)
Postoperative CT	
Yes	8 (16.0)
No	42 (84.0)
pT stage	
pT1–2	25 (50.0)
pT3–4	25 (50.0)
pN stage	
pN0	30 (60.0)
pN1–3	20 (40.0)
Pathological staging	
I/II	17 (34.0)
III/IV	33 (66.0)

RT, radiotherapy; CT, chemotherapy; OSCC, oral squamous cell carcinoma.

**Table II:** Concentration of MIF in the pre- and postoperative serum and saliva of oral squamous cell carcinoma patients and its association with disease outcome.

		Preoperative serum MIF (ng/ml)			Postoperative serum MIF (ng/ml)				Preoperative saliva MIF (ng/ml)			Postoperative saliva MIF (ng/ml)		
										
Variable	n	Range	Median	n	Range	Median	P-value^a^	n	Range	Median	n	Range	Median	P-value^a^
Local recurrence														
No	34	17.7–136.6	53.2	28	16.2–118.5	35.2	**0.014**	33	42.7–1059.1	248.7	27	79.7–1015.5	244.0	0.732
Yes	12	17.7–95.0	46.1	10	15.1–55.3	28.8	**0.012**	12	32.5–1770.6	468.0	8	64.5–2046.8	342.9	0.575
P-value			0.423			0.208				0.072			0.239	
Regional recurrence														
No	39	17.7–136.6	51.1	32	15.1–118.5	34.9	**0.004**	38	42.7–1770.6	320.8	30	79.7–2046.8	256.1	0.854
Yes	7	20.3–95.5	48.6	6	23.2–35.3	27.8	**0.046**	7	32.5–1013.2	499.0	5	64.5–543.5	261.6	0.500
P-value			0.963			0.149				0.900			0.741	
Distant metastasis														
No	44	17.7–136.6	49.8	36	15.1–118.5	33.7	**0.002**	43	32.5–1770.6	282.4	33	64.5–2046.8	250.9	0.896
Yes	2	59.6–95.5	77.6	2	30.7–35.3	33.0	NE	2	461.8–764.3	613.0	2	278.4–512.1	395.2	NE
P-value			0.161			0.999				0.247			0.286	
Local or regional recurrence														
No	31	17.7–136.6	52.3	26	16.2–118.5	35.7	**0.021**	30	42.7–1059.1	228.8	25	79.7–1015.5	244.0	0.257
Yes	15	17.7–95.5	48.3	12	15.1–55.3	28.8	**0.010**	15	32.5–1770.6	474.2	10	64.5–2046.8	279.5	0.799
P-value			0.582			0.149				0.060			0.342	
Second primary tumor														
No	40	17.7–136.6	49.8	32	15.1–118.5	34.4	**0.006**	39	42.7–1770.6	332.2	29	79.7–1015.5	261.4	0.699
Yes	6	17.7–104.6	59.5	6	23.2–41.5	31.4	0.075	6	32.5–960.3	300.8	6	64.5–2046.8	261.2	0.249
P-value			0.514			0.603				0.664			0.965	
Mortality from cancer														
No	37	17.7–136.6	50.1	32	15.1–118.5	34.9	**0.004**	36	42.7–1490.2	228.8	31	79.7–1015.5	250.9	0.861
Yes	9	17.7–95.5	59.6	6	23.2–35.3	28.8	0.075	9	32.5–1770.6	764.3	4	64.5–2046.8	395.2	0.465
P-value			0.589			0.200			**0.023**				0.437	

Bold P-value indicates statistical significance. P-value obtained from nonparametric Mann-Whitney U test, unless specified otherwise.

a Obtained from Wilcoxon signed rank test for matched samples.

MIF, macrophage migration inhibiroty factor; NE, not evaluable.

**Table III:** Concentration of MIF in pre- and postoperative serum and saliva in patients with oral squamous cell carcinoma and its association with clinicopathological variables.

		Preoperative serum MIF (ng/ml)				Postoperative serum MIF (ng/ml)				Preoperative saliva MIF (ng/ml)				Postoperative saliva MIF (ng/ml)		
												
Variable	n	Range	Median	P-value	n	Range	Median	P-value	n	Range	Median	P-value	n	Range	Median	P-value
Age (years)				0.051				0.826				0.992				0.594
≤56.5	25	17.7–95.5	44.0		21	15.2–78.1	33.2		25	32.5–1013.2	348.5		20	64.5–2046.8	270.0	
>56.5	25	20.9–136.6	59.1		17	16.2–118.5	34.2		23	67.6–1770.6	309.4		15	122.1–1015.5	244.0	
Smoking status				0.711				0.216				0.072				**0.040**
Current	42	17.7–136.6	51.7		34	15.1–118.5	32.7		40	32.5–1770.6	228.8		29	64.5–2046.8	244.0	
Ex-smoker	8	25.8–76.2	49.8		4	32.4–45.6	40.9		8	82.6–1059.1	466.5		6	221.8–1015.5	373.6	
Alcohol consumption				0.630				0.263				0.587				0.919
Current	29	17.7–104.6	50.1		24	15.1–118.5	31.6		28	32.5–1490.2	216.2		21	64.5–1015.5	261.4	
Never or ex-drinker	21	17.7–136.6	56.9		14	20.5–78.1	35.2		20	42.7–1770.6	383.6		14	106.5–2046.8	238.6	
pT stage				0.184				0.619				**0.001**				0.091
pT1 + pT2	25	17.7–136.6	57.0		18	16.2–118.5	35.8		24	45.6–867.6	165.9		19	79.7–878.6	215.8	
pT3 + pT4	25	17.7–95.0	48.3		20	15.1–78.1	32.8		24	32.5–1770.6	445.8		16	64.5–2046.8	314.7	
Lymph node status (pN)				**0.018**				0.362				0.317				0.570
pN0	30	20.9–136.6	56.9		23	16.2–118.5	36.3		29	42.7–1490.2	255.5		24	79.7–1015.5	261.4	
pN+	20	17.7–104.6	37.8		15	15.1–74.8	32.3		19	32.5–1770.6	474.2		11	64.5–2046.8	250.9	
Pathological staging				**0.040**				0.380				**0.032**				0.479
GI + GII	17	20.9–136.6	59.6		13	16.2–118.5	36.9		17	45.6–461.8	172.6		14	79.7–878.6	229.9	
GIII + GIV	33	17.7–104.6	48.3		25	15.1–78.1	32.4		31	32.5–1770.6	417.4		21	64.5–2046.8	261.6	
Differentiation grade				0.618^a^				0.997^a^				0.938^a^				0.883^a^
Poor	2	17.7–104.6	61.2		2	28.1–41.5	34.8		2	67.6–960.3	514.0		2	122.1–2046.8	1084.5	
Moderate	38	20.3–136.6	51.7		31	15.1–118.5	34.2		36	32.5–1059.1	320.8		27	64.5–1015.5	261.4	
Well	10	17.7–79.1	44.3		5	18.3–51.0	32.3		10	82.6–1770.6	254.9		6	106.5–832.8	261.6	
Blood vessel invasion				0.562				NE				0.700				0.815
Not detected	45	17.7–129.5	48.6		34	15.1–118.5	32.3		43	32.5–1770.6	255.5		30	64.5–2046.8	261.5	
Detected	2	56.9–59.6	58.2		1	78.1	78.1		2	348.5–406.2	377.4		2	221.8–261.4	241.6	
Lymphatic invasion				0.960				0.724				0.653				0.079
Not detected	36	17.7–129.5	49.8		27	15.1–118.5	32.4		34	42.7–1490.2	252.1		25	79.7–2046.8	272.8	
Detected	11	22.8–104.6	48.6		8	23.2–78.1	33.4		11	32.5–1770.6	406.2		7	64.5–278.4	221.8	
Perineural invasion				0.714				0.711				0.818				0.258
Not detected	27	17.7–129.5	49.5		21	15.1–118.5	32.4		26	49.1–1059.1	252.1		21	112.8–2046.8	261.4	
Detected	20	22.8–95.0	49.9		14	19.9–78.1	33.4		19	32.5–1770.6	406.2		11	64.5–832.8	261.4	
Inflammatory infiltrate				0.510				0.338				0.988				0.872
Poor	12	17.7–95.0	44.0		8	15.1–55.3	31.2		11	42.7–1770.6	176.9		6	79.7–2046.8	275.4	
Moderate + intense	29	20.3–129.5	49.5		24	16.2–118.5	33.9		29	32.5–1490.2	332.2		23	64.5–1011.9	261.4	
Surgical margin status				0.355				0.645				**0.045**				0.887
Tumor-free	44	17.7–136.6	50.6		35	15.1–118.5	34.2		43	32.5–1490.2	248.7		33	64.5–2046.8	261.4	
Positive	6	39.0–95.0	58.2		3	29.5–55.3	33.2		5	309.4–1770.6	499.0		2	150.0–543.5	346.7	

Bold P-value indicates statistical significance. P-value obtained from nonparametric Mann-Whitney U test, unless specified otherwise.

a Obtained from nonparametric Kruskal-Wallis test.

MIF, macrophage migration inhibitory factor; NE; not evaluable.

**Table IV:** Corrleation between the concentration of MIF in pre- and postoperative serum and saliva samples of oral squamous cell carcinoma and tumor characteristics.

		Preoperative serum MIF (ng/ml)				Postoperative serum MIF (ng/ml)				Preoperative saliva MIF (ng/ml)				Postoperative saliva MIF (ng/ml)		
												
Variable	n	Range	Median	P-value	n	Range	Median	P-value	n	Range	Median	P-value	n	Range	Median	P-value
Lymph node involvement				**0.023**				0.922				0.468				0.347
No	28	20.9–129.5	55.5		22	15.1–118.5	32.8		27	42.7–1490.2	255.5		23	79.7–1015.5	261.4	
Yes	20	17.7–104.6	37.8		14	23.2–74.8	33.2		19	32.5–1770.6	474.2		10	64.5–2046.8	236.3	
Largest diameter of metastatic lymph node (cm)				0.545				0.072				0.327				0.425
≤1.5	10	17.7–59.6	35.8		5	29.5–74.8	35.2		10	76.4–1770.6	426.2		3	221.8–388.9	250.9	
>1.5	10	17.7–104.6	40.1		9	23.2–41.5	28.2		9	32.5–960.3	474.2		7	64.5–2046.8	162.3	
Lymph node index				**0.025**				0.338				0.447				0.174
≤3.5	12	17.7–59.6	26.7		7	24.0–74.8	34.6		12	49.1–1770.6	426.2		5	162.3–2046.8	250.9	
>3.5	8	23.1–104.6	56.7		7	23.2–41.5	29.5		7	32.5–1013.2	474.2		5	64.5–278.4	122.1	
Largest diameter of primary tumor (cm)				0.754				0.724				**0.020**				**0.028**
≤2.5	13	17.7–104.6	54.2		10	18.3–118.5	33.8		13	49.1–867.6	125.0		10	79.7–512.1	166.5	
>2.5	35	17.7–129.5	48.6		26	15.2–78.1	32.8		33	32.5–1770.6	406.2		23	64.5–2046.8	278.4	
General index				0.071				0.648				**0.025**				0.120
≤2.9	10	20.9–99.9	64.4		8	18.3–118.5	33.8		10	45.6–461.8	142.0		8	79.7–512.1	193.2	
>2.9	38	17.7–129.5	46.1		28	15.1–78.1	32.8		36	32.5–1770.6	395.5		25	64.5–2046.8	261.6	

Bold P-value indicates statistical significance. P-value obtained from nonparametric Mann-Whitney U test, unless specified otherwise. MIF, macrophage migration inhibitory factor.
